# Perceived barriers and learning anxiety in online education among domestic and international students

**DOI:** 10.3389/fpsyg.2026.1685494

**Published:** 2026-02-16

**Authors:** Chen Yu

**Affiliations:** College of International Education and Social Development, Zhejiang Normal University, Jinhua, Zhejiang, China

**Keywords:** anxiety, barriers, COVID-19, educational technology, international students, online learning

## Abstract

Due to the COVID-19 pandemic, university students in China experienced a rapid shift to online education beginning in late 2019. However, online learning experiences differed substantially between domestic students, who underwent hybrid instruction with repeated transitions between online and in-person classes, and international students, many of whom experienced fully online instruction for more than 2 years. Grounded in Learning Anxiety Theory and the Technological Pedagogical Content Knowledge (TPACK) framework, this study developed an Online Learning Anxiety State Scale (Cronbach’s α = 0.780) and an Online Education Obstacle scale (Cronbach’s *α* = 0.779), which demonstrated acceptable overall reliability and validity. Using these instruments, the study examined perceived barriers to online learning-particularly online teaching skills and technological factors-and their relationships with online learning anxiety among domestic (*N* = 133) and international (*N* = 86) undergraduate students enrolled in Chinese universities. Results identified three primary dimensions-teachers’ teaching skills, internet/equipment conditions, and environmental factors-as significant contributors to heightened online learning anxiety. These factors were significantly associated with students’ anxiety levels in online learning contexts. Furthermore, notable group differences emerged: compared with domestic students, international students perceived environmental factors as more salient sources of anxiety than internet or equipment-related barriers. This study fills a key gap by examining how multiple online learning obstacles relate to anxiety, comparing domestic and international students. Integrating Equity Theory and Expectancy-Value Theory, it reconceptualizes online learning anxiety as a multifaceted psychological response shaped by fairness perceptions and unmet expectations. Findings advance theory, guide post-pandemic higher education toward modality equity, and inform equitable, psychologically sustainable distance and blended learning design.

## 1 Introduction

Due to the COVID-19 pandemic, university students and faculty worldwide underwent an abrupt, large-scale transition to online and hybrid education in late 2019 and early 2020, distinct from pre-pandemic voluntary online learning models. Unlike prior opt-in virtual learners, many pandemic-era students were compelled to engage in online/blended instruction due to external constraints, with experiences ranging from temporary hybrid arrangements to prolonged fully online learning (Fawaz and Samaha, 2021; Phanphech et al., 2022). Despite decades of online education integration in higher education, persistent barriers remain, including suboptimal instructional design, self-directed learning challenges, and technological limitations (Muilenburg and Berge, 2005; O’Doherty et al., 2018). This study investigates perceived technology-education integration barriers (teaching skills, technological factors) and online learning anxiety among domestic and international undergraduates at Chinese universities, focusing on this pandemic-induced mandatory online learning context.

Barriers to technology integration in higher education have been documented since the advent of educational technology, encompassing restricted hardware/software access, insufficient technical training/support, low digital literacy, negative stakeholder attitudes, and inadequate institutional resources/policies (Hoang et al., 2019; Mayer, 2019; Razaghi, 2014; Rogers, 2000; Stuart et al., 2009; Wong et al., 2019). Researchers classify these as internal (cognitive/attitudinal factors: attitudes, self-efficacy, digital skills, cultural perceptions; Hoang et al., 2019) and external (systemic constraints: equipment deficits, poor connectivity, limited support, financial/policy barriers; Alqurashi, 2016), with dynamic interactions between the two-e.g., course environments may mediate the attitude-anxiety link (Zhao, 2022). Multi-dimensional measurement is essential given the divergent solutions required for distinct barrier categories.

Technology anxiety, defined as uncertainty/fear toward computing systems leading to avoidant behavior (Nguyen et al., 2019), manifests uniquely in online learning (Ajmal and Ahmad, 2019). Students with limited digital experience are particularly vulnerable (Abdous, 2019), and such anxiety impairs engagement and performance (Stan et al., 2022; Zapata-Cuervo et al., 2023). Pandemic-era online learning anxiety surged across synchronous/asynchronous modalities (Fawaz and Samaha, 2021; Phanphech et al., 2022). In China, domestic undergraduates had intermittent online learning (2020–2022), while international students faced up to five semesters of fully online instruction due to travel restrictions lifted in April 2022, coinciding with a one-third decline in international student enrollment by June 2022 (Guangming Online, 2022).

The pandemic-driven shift to fully online instruction transformed Chinese higher education, highlighting the urgent need to examine barrier-anxiety interplay in this context. Prior research links technology integration barriers, technology anxiety, and language learning anxiety to online language learning outcomes (Ajmal and Ahmad, 2019; Bolliger and Halupa, 2012), yet gaps exist in cross-group comparisons between domestic and international students in China’s pandemic context.

This study explores similarities/differences in their perceived technology-education integration barriers and online learning anxiety, addressing three research questions: (1) Did perceived barriers to online learning and online learning anxiety differ between domestic and international students studying at Chinese universities during COVID-19? (2) How did perceived barriers to online education relate to anxiety among students studying at Chinese universities during COVID-19? (3) Were the relations between perceived barriers and anxiety different between domestic and international students? Findings contribute to understanding diverse learner challenges in emergency remote education, informing inclusive post-pandemic online learning environments.

## 2 Research method and process

### 2.1 Measurement instruments

Two instruments were developed for this study: the Online Learning Anxiety State Scale (hereafter referred to as the anxiety scale) and the Online Education Obstacle scale (hereafter referred to as the obstacle scale).

The anxiety scale was adapted from the State–Trait Anxiety Inventory (STAI) with wording contextualized to online learning, while the obstacle scale was constructed based on the Technological Pedagogical Content Knowledge (TPACK) framework and relevant prior literature (Mishra and Koehler, 2006; Stuart et al., 2009; Razaghi, 2014; Hoang et al., 2019; Kouser and Popat, 2022).

Item generation was informed by preliminary qualitative investigation and expert review. The final Anxiety Scale comprised 13 items (Cronbach’s α = 0.780), and the final obstacle scale (Cronbach’s α = 0.779) consisted of 20 items (out of original 28 items) across five dimensions. From Table 1, it can be seen that the five dimension is: teacher skill factors, internet/equipment factors, environmental factors, teaching material and interactional factors. (see Table 1 for more details).

**Table 1:** Internal consistency test results of all dimensions of Integration obstacle scale.

Dimension	Cronbach’s *α*
Teacher skill factors	0.827
Internet/equipment factors	0.844
Environmental factors	0.695
Teaching materials	0.613
Interactional factors	0.414

All items used a 5-point Likert scale (1 = strongly disagree to 5 = strongly agree). The anxiety scale consists of seven positively keyed items and six negatively keyed items; the obstacle scale consists of 11 positively keyed items and nine negatively keyed items.

Both scales were validated via Exploratory Factor Analysis (EFA) (see Supplementary Tables 1a–c), with the latter demonstrating satisfactory model fit. Inter-item reliability for the subscales of the Obstacle scale, along with detailed confirmatory factor analysis (CFA) fit indices, is reported in Table 2.

**Table 2:** Model fit index (CFA result for obstacle scale).

Common indicators	χ^2^	*df*	(χ^2^/*df*)	GFI	RMSEA	RMR	CFI	NFI	NNFI
Value	352.428	160	2.203	0.865	0.074	0.108	0.737	0.897	0.852

Full protocols for scale development, translation, and item reduction procedures are also provided in the Supplementary material.

### 2.2 Participants

A total of 219 undergraduate students (133 domestic students and 86 international students; 143 women and 76 men; ages 18–28) were recruited from three Chinese universities; 212 (132 were from Chinese undergraduates and 80 from international students) valid questionnaires were collected (characterized by answer times less than 90 s, random filling in of answers, uniform answer options, or answers with regular patterns). The students from these three universities were chosen as the research sample because all three universities are located in the same province (One of the most complete online teaching platform construction provinces in China). During the COVID-19 pandemic, these institutions implemented identical online teaching policies and adopted the same learning platforms. Limiting the sample to this region eliminates confounding effects arising from cross-regional discrepancies in policies (e.g., requirements for course duration) and divergences in the functionalities of online teaching platforms (e.g., interactive tools and technical stability).

### 2.3 Data processing

Links to the study measures were shared with students by their university professors. Data collection was carried out via “Questionnaire Star.” Data collection for the study occurred from April–June 2022. All data was analyzed using the SPSS 26, SPSSAU and Excel 2016.

### 2.4 Analysis

The analysis primarily focuses on the obstacle scale, aiming to examine the different dimensions of barriers to technology-education integration and their potential impact on online learning anxiety. Item analysis and factor analysis were conducted to assess structural validity. Of the 219 questionnaires collected in 2022, 212 were retained after excluding invalid responses, yielding an effective response rate of approximately 97%.

#### 2.4.1 Item analysis

The purpose of item analysis is to assess the degree of differentiation among items on the scale. Initially, SPSS 26 was utilized to sum all the items of the obstacle scale. Subsequently, the lowest 27% of the subjects (scoring 73 or below), were categorized into a low group; and the highest 27% of the subjects (scoring 87 or above), were categorized into a high group. In the third step, an independent samples *t*-test was conducted to evaluate the significant difference between the high and low groups. The results of Levene’s test for variance equality, revealed that *t* = −21.639, *p* ≈ 0.000 < 0.01, indicating that the scores of the high and low groups were significantly different. This suggests that the obstacle scale has a good degree of differentiation.

#### 2.4.2 Exploratory factor analysis of the obstacle scale

Factor analysis is to simplify and revise the items under the corresponding dimension. In this study, SPSS 26 was used to analyze the structural validity of the original 28 items through exploratory factor analysis (EFA). The KMO index, which represents the sampling adequacy in EFA, had a value of 0.896, and Bartlett’s test of sphericity yielded a statistic of 2435.795, with *p* = 0.000, indicating that the data is suitable for factor analysis (more details please refer to Supplementary Tables 1a–c). The Promax with Kaiser Normalization in the principal component method is used to extract factors with factor loadings greater than 1.

#### 2.4.3 Reliability analysis

SPSS 26 was used to test the internal consistency of the five factors of the obstacle scale, and the results were shown in Table 1. As shown in Table 1, the internal consistency of each dimension of the obstacle scale was assessed using Cronbach’s α in this exploratory study. The five dimensions yielded a coefficient ranging from 0.414 to 0.844. The internet/equipment factors dimension demonstrated the highest reliability (*α* = 0.844), followed by the skill factors dimension (*α* = 0.827). Both exceeded the 0.80 threshold, indicating excellent internal consistency. The environmental factors dimension (*α* = 0.695) and the teaching materials dimension (0.613) showed acceptable reliability.

In contrast, the interactional factors dimension exhibited relatively low internal consistency (*α* = 0.414). It should be noted that Cronbach’s α assumes unidimensionality. However, the present scale was designed to capture obstacles to online learning during forced online instruction amid the COVID-19 pandemic. Given the inherently multidimensional nature of online learning contexts and anxiety-related experiences, a coefficient may underestimate reliability when applied to relatively heterogeneous or independent dimensions.

Moreover, this study is exploratory, aiming to identify potential factors influencing online learning anxiety rather than to establish a finalized psychometric instrument. Accordingly, the interactional factors dimension was excluded from core inferential analyses and retained solely for exploratory and descriptive purposes. Notably, lower Cronbach’s alpha values are not only acceptable but also theoretically expected in such exploratory contexts (e.g., Nunnally and Bernstein, 1994; Tavakol and Dennick, 2011). Specifically, Nunnally and Bernstein (1994) argue that in the early stages of research-where the primary goal is construct exploration rather than confirmatory inference-reliability coefficients in the range of 0.40 to 0.50 may be tolerated, as the focus lies on identifying potential construct domains rather than establishing robust measurement for definitive hypothesis testing. As a newly developed subscale, it should therefore be regarded as provisional. In addition, at the scale level, the full 20-item instrument yielded a Cronbach’s α of 0.779, indicating acceptable overall internal consistency for exploratory research.

From Supplementary Table 1a, it can be observed that the items are screened one by one based on three main criteria: items were deleted because of lower than factor loadings of 0.5; items were deleted because of common factor variance value less than 0.5; and items were deleted because that there is a serious deviation in the corresponding relationship between item and factor. Finally, a problem book comprising 5 factors and 20 items is formed.

From the Supplementary Table 1c, we can see that the interpretive variance of the five factors for the total score of the obstacle was approximately 61.259%. This indicates that the five-dimensional construct validity of the obstacle scale proposed by us has been preliminarily verified. The correlation between each item and the total scale ranged from 0.148 to 0.758 (*p* < 0.01), and the correlations between the five subscales and the total scale were 0.758, 0.675, 0.663, 0.496, and 0.148 (*p* < 0.01). According to the main contents of the items under each factor, the factors are named as follows: Factor 1 (teacher skill factor) with 5 questions, Factor 2 (network equipment factor) with 5 questions, Factor 3 (environmental factor) with 4 questions, Factor 4 (teaching material factor) with 3 questions, and Factor 5 (interactive factor) with 3 questions (see Supplementary Table 1a).

#### 2.4.4 Confirmatory factor analysis of the obstacle scale

SPSSAU (an intelligent online statistical analysis platform, and its website is https://spssau.com/index.html) was used to apply the confirmatory factor analysis (CFA) of the obstacle scale. As presented in the Table 2, the results of CFA indicated an acceptable but suboptimal model fit (*χ*^2^/df = 2.203 < 3, RMSEA = 0.074 < 0.1). Although the *χ*^2^/df ratio and RMSEA met commonly accepted thresholds, incremental fit indices such as CFI (0.737) and NNFI (0.852) did not meet the recommended standards for good fit, which further confirms, to some extent, the potential impact of the low reliability of the interactional factor observed in the reliability analysis. These fit indices collectively demonstrate an acceptable overall model fit, consistent with common psychometric criteria for structural equation modeling (Pan and Liu, 2024).

## 3 Results

As expected, the amount of time spent in online learning was longer for international students than for domestic Chinese students (*t* = −3.772, *p* ≈ 0.0002 < 0.001).

**Research question 1.** Did perceived barriers to online learning and online learning anxiety differ between domestic and international students studying at Chinese universities during COVID-19?

By nationality. A comparative analysis of perceived barriers to technology-education integration by nationality (Supplementary Table 2a) indicates modest differences between Chinese and international undergraduates.

First, both groups identified teacher skills, internet/equipment, and environmental factors as the three most salient obstacles, and consistently ranked teaching materials as the least significant obstacle. However, Chinese students rated environmental factors as more problematic than network barriers, whereas international students perceived internet/equipment constraints as more prominent than environmental factors.

Additionally, Chinese undergraduates considered interactional barriers to be as serious as internet/equipment barriers, while international students ranked interactional barriers fourth.

Results of independent-samples *t*-tests (Supplementary Table 2a) showed that international students reported significantly greater internet/equipment barriers than Chinese students (*t* = −7.255, *p* < 0.01), whereas Chinese students reported significantly higher interactional barriers than international students (t = 2.558, *p* = 0.011 < 0.05).

By IP. A comparative analysis of barriers to technology-education integration between overseas and domestic undergraduates based on IP location shows significant differences between the groups, except for interaction factors (Supplementary Table 2a).

Independent-samples t-tests indicate that overseas students reported higher obstacles in teaching skills (*t* = −2.624, *p* = 0.009), internet/equipment factors (*t* = −6.649, *p* < 0.001), environmental factors (*t* = −3.478, *p* = 0.001), and teaching materials (*t*= − 2.036, *p* = 0.045).

Moreover, online learning anxiety was significantly greater in international students than in domestic students, whether categorized by nationality (*t* = −3.837, *p* ≈ 0.0002) or by IP (*t* = −5.393, *p* < 0.01). See Supplementary Table 2a for further details.

**Research question 2.** How did perceived barriers to online education relate to anxiety among students studying at Chinese universities during COVID-19?

Anxiety was significantly correlated with all obstacle factors except the interaction factor (Supplementary Table 1b), indicating that higher perceived obstacles are associated with greater online learning anxiety. To examine which factors predict anxiety, linear regression was conducted (Supplementary Table 1c).

Using student anxiety as the dependent variable, sequential regression showed that adding the anxiety factor to the original model-including teaching skill, technological, and environmental factors-significantly improved the model. The final model (Model 3) was significant, with *R*^2^ = 0.531, adjusted *R*^2^ = 0.520, and Δ*R*^2^ = 0.039, demonstrating that these obstacles collectively account for a substantial portion of variance in online learning anxiety.

**Figure 1:**
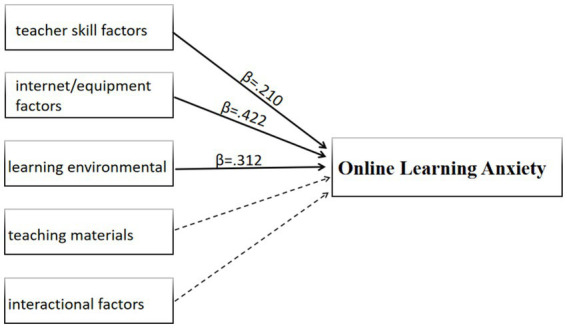
Conceptual diagram illustrating online anxiety and related obstacles.

From Supplementary Table 1c, internet/equipment factors, environmental factors and teacher skill factors had the strongest positive effects on online learning anxiety, and also exhibited a significant positive effect. In contrast, teaching materials and interactional factors were positively associated with online anxiety but did not reach statistical significance. These findings suggest that technological and environmental barriers play a more prominent role in predicting students’ online learning anxiety than teaching materials or interactional challenges. Based on these results, the conceptual diagram illustrating online anxiety and related obstacles is presented in Figure 1.

**Research question 3.** Were the relations between perceived barriers and anxiety different between domestic and international students?

Correlation analyses for relations between obstacles and anxiety separated by domestic/international status are shown in Supplementary Table 3a. From Supplementary Table 3a, it can be seen that for domestic students, anxiety was strongly correlated with teacher skill factors (*r* = 0.597, *p* < 0.01) and environmental factors (*r* = 0.577, *p* < 0.01), moderately correlated with internet/equipment factors (*r* = 0.333, *p* < 0.01) and teaching materials (*r* = 0.261, *p* < 0.01). For international students, anxiety was most strongly correlated with internet/equipment factors (*r* = 0.649, *p* < 0.01), followed by teaching materials (*r* = 0.422, *p* < 0.01), environmental factors (*r* = 0.371, *p* < 0.01), and teacher skill factors (*r* = 0.322, *p* < 0.01).

These findings indicate that teacher skill and environmental barriers are more salient for domestic students, whereas internet/equipment barriers are more salient for international students.

## 4 Discussion

Through the investigation of barriers to the integration of online technology and education and their relation to online learning anxiety, two scales with good differentiation, reliability and construct validity were developed. A central finding is that, for both Chinese and international students, teachers’ teaching skills represent the most significant obstacle to effective online education. This highlights a widespread deficiency in teachers’ information literacy, which encompasses the ability to integrate information technology with teaching, including information awareness, knowledge, ethics, and the capacity to design and deliver content effectively using online platforms. High information literacy enables teachers to align course content with platform functionalities, design and organize online activities according to class objectives, facilitate real-time interaction, and manage Q&A and discussion efficiently.

The lack of information literacy among teachers manifests primarily in three areas: voice and sound quality during online sessions, proficiency in operating software or platform functions, and the ability to convey information effectively. Many teachers are unfamiliar with online management systems, live streaming tools, and platform features, leading to inefficient content delivery, delayed interactions, and reduced engagement. These challenges were further compounded by the sudden shift to online teaching during the COVID-19 pandemic, which forced educators-including those inexperienced with or resistant to digital tools-into fully online classrooms. Teachers accustomed to traditional methods often experienced emotional reluctance or resistance to online teaching, further limiting their adoption of digital strategies and reducing the effectiveness of online instruction. The psychological adjustment required for this rapid transition, coupled with insufficient preparation in hardware and technical resources, created additional barriers. Many educators lacked professional-grade equipment such as noise-canceling microphones, relying instead on standard headphones, which compromised sound quality. Similarly, unfamiliarity with online teaching software and tools, coupled with limited strategies for classroom management in virtual environments, reduced the overall effectiveness of online instruction. Wu et al. (2020) note that these deficits in teacher readiness contributed to suboptimal online teaching experiences during the pandemic, highlighting the critical role of technical competence and digital adaptability in modern education. The findings suggest that improving teachers’ information literacy and technical competence can substantially mitigate perceived obstacles, thereby reducing anxiety and enhancing the effectiveness of online instruction.

The analysis further reveals that while teaching skills are the most significant barrier, other factors such as internet/equipment reliability and environmental conditions also contribute greatly to students’ perceived obstacles. For instance, inadequate hardware or unstable network connections directly impact content delivery and interaction quality. Together, these barriers interact with teacher skills to shape students’ overall online learning experiences and influence levels of online anxiety.

From a theoretical perspective, these results underscore the importance of integrating teacher readiness and digital competence into models of online learning efficacy. The concept of information literacy provides a useful framework for understanding how educators’ technological capabilities interact with structural and environmental factors to affect learning outcomes.

Practically, the findings highlight several actionable recommendations. First, professional development programs should prioritize enhancing teachers’ information literacy, focusing on digital content design, platform navigation, and interactive teaching strategies. Second, institutions should ensure that educators have access to adequate hardware and reliable network resources to support high-quality online instruction. Third, psychological support and gradual acclimatization to online teaching may help teachers overcome emotional resistance and increase their confidence in digital environments. Collectively, these measures can address both technical and affective dimensions of teacher preparedness, thereby improving student experiences and reducing anxiety associated with online learning.

Secondly, beyond teachers’ online teaching skills, internet/equipment conditions emerged as one of the three most significant barriers to online teaching, corroborating previous findings (Alshwiah, 2021; Unger and Meiran, 2020; Ubogu and Orighofori, 2020). Notably, technological convenience-commonly regarded as an advantage of online learning-was perceived by undergraduates as an obstacle, suggesting that current infrastructures remain insufficient to support long-term, large-scale online education.

For international students, network/equipment barriers ranked even higher, particularly among those studying overseas. When learners were grouped by IP address, overseas students reported significantly greater technological constraints than domestic students, indicating persistent disparities in network infrastructure between China and other regions. These barriers were perceived as more salient than environmental factors, regardless of students’ physical location.

Qualitative evidence suggests that these challenges are closely tied to socioeconomic and infrastructural conditions. Many international students, particularly those from African and Southwest Asian countries, face high internet costs, unstable connections, and limited access to appropriate digital devices. Such constraints often result in interrupted participation, reduced access to learning materials, and reliance on partial downloads or mobile-only learning, all of which are likely to exacerbate learning anxiety.

From a practical perspective, network instability, platform congestion, and device limitations frequently disrupt instructional flow and interaction, undermine timely feedback, and complicate classroom management. These findings underscore the need for institutions to address technological inequities by improving platform stability, optimizing low-bandwidth instructional designs, and providing targeted technical and financial support for international students.

Thirdly, environmental factors emerged as one of the top three barriers to online learning, with a subtle difference in ranking between Chinese and international students. International students placed environmental factors below network/device barriers, whereas Chinese students ranked them higher. This discrepancy may reflect contextual differences: Chinese undergraduates typically take more courses and accrue higher credit loads than international students, contributing to greater physical and mental exhaustion. Additionally, strict domestic COVID-19 measures, such as frequent testing, increased stress and reduced concentration among Chinese students. Both groups reported that prolonged online sessions exacerbate physical discomfort, impair focus, and reduce patience with assignments, suggesting that environmental challenges interact with mental strain and task demands to influence students’ online learning experiences.

The fourth dimension, instructional materials, was identified as a minor but relevant barrier to online learning. Findings suggest that online platforms often do not provide substantially more resources than traditional classrooms, limiting students’ opportunities for repeated self-directed learning. Many teachers either lack the technical skills or are reluctant to record and share video lessons due to concerns about students’ internet access, intellectual property rights, and platform restrictions. Consequently, online courses often function primarily as tools for information delivery rather than as enriched learning environments. Similarly, the shift from paper-based to electronic assignment submission increases procedural complexity, adding to students’ perceived burden.

While four obstacle factors were positively correlated with learners’ online learning anxiety, only teacher skills, internet/equipment, and environmental factors showed strong predictive effects. Lower teaching proficiency, limited technological resources, or suboptimal physical and mental conditions among learners corresponded to higher anxiety.

Furthermore, the amount of online learning time also influenced anxiety in a nonlinear fashion. During the initial 6 months of forced online instruction, such as during the COVID-19 pandemic, learners’ anxiety gradually increased. This trend aligns with broader evidence that large-scale social emergencies elevate public anxiety (Liu and Li, 2021; Alshwiah, 2021; Taylor et al., 2020), indicating that learners’ emotional responses reflect both the challenges of online instruction and the psychological impact of societal crises. Therefore, learner anxiety is shaped not only by instructional barriers but also by broader contextual and temporal factors, emphasizing the interplay between educational and environmental conditions in online learning contexts.

After half a year to about a year of online teaching, the anxiety was reduced. This may be because this period is the peak period of the global epidemic. On the one hand, everyone is gradually adapting to this environment. On the other hand, students find that their classmates are quarantining at home and studying online, and even working family members are working online. Experiences between social groups are similar with minimal differences, so anxiety gradually decreases. But after that, when online teaching methods are continuously used for a cumulative period of about 18 months or more, learners’ online anxiety will rise sharply. This may be because the global epidemic has changed accordingly at this time, and the epidemic prevention measures and policies of various countries have begun to differ. There is a contrast between groups, between societies, and even between countries. This psychological change caused by difference and contrast leads to anxiety in learners. This view is also supported by our survey data: 74.82% of students rejected the scenario in which their own classes were fully online while peers attended in-person courses. This strong preference for instructional equity aligns with Equity Theory (Li et al., 2025), which suggests that perceived disparities in instructional modes create a sense of procedural injustice that may exacerbate online learning anxiety. These findings extend prior research by highlighting that online learning anxiety is not solely a technological issue, but also a psychological response related to fairness perceptions.

Furthermore, the observed differences in online learning anxiety between domestic and international students can be partially explained through this fairness lens. Due to objective differences in network and device conditions, students may perceive imbalances in instructional access, which can be interpreted as unfair allocation of educational resources, thereby exacerbating anxiety. This suggests that, in online teaching practice, schools and educational administrators should consider potential fairness issues arising from differences in network and device conditions, and their possible impact on students’ emotional and psychological experiences.

In addition, domestic undergraduates faced a different set of anxiety drivers. Their four-year degree programs overlapped with China’ s three-year pandemic response, characterized by repeated and often abrupt shifts between online, hybrid, and offline instruction. This instability created a mismatch between students’ expectations of continuous in-person education and the reality of fragmented learning arrangements. Such expectation-outcome discrepancies align with Expectancy-Value Theory (Vo and Ho, 2024): when expected learning conditions are unmet, the perceived value of online learning decreases, thereby increasing anxiety.

These findings mentioned above have important implications for post-pandemic higher education globally. First, ensuring modality equity is critical for transnational student populations; universities with large international enrollments should avoid asynchronous or unequal modality arrangements that may induce fairness-related anxiety. Second, evidence-based limits on online learning-such as avoiding ad-hoc transitions and ensuring intentional, well-supported technology integration-can help prevent frustration of core psychological needs. Third, targeted institutional support for international students-especially in terms of network infrastructure and access to appropriate learning equipment-may mitigate learning anxiety by reducing perceived inequities in instructional conditions and promoting a more equitable online learning environment.

Overall, this study advances theory-driven explanations of online learning anxiety and offers globally relevant guidance for designing equitable and psychologically sustainable hybrid education models.

## 5 Limitations and outlook

Two years after the end of China’s epidemic prevention and control policy, the anxiety of international students about online learning has been alleviated, while the anxiety of Chinese undergraduates about online learning has increased instead of decreasing (according to the results of our latest follow-up experiment in 2024). This seems to remind us that we still need to pay attention to the barriers to the integration of technology and education in future teaching, because hybrid teaching is the trend of education in the future. It is also inevitable that technology will be used to supplement teaching in daily teaching. With the development of AI, existing research has largely emphasized its benefits for teaching and assessment. However, the psychological implications of AI-mediated instruction-particularly in conventional face-to-face or hybrid classroom settings-remain remain underexplored, even though technology-related anxiety has been widely discussed in other contexts.

In addition, as the international student sample was predominantly drawn from Africa and Asia, with relatively limited representation from America and Europe, caution is warranted when generalizing the findings to international student populations from other regions.

## Data Availability

The raw data supporting the conclusions of this article will be made available by the authors, without undue reservation.
