# Supplementary material for: Perceived barriers and learning anxiety in online education among domestic and international students
Source: Front Psychol. 2026 Feb 16;17:1685494. doi: 10.3389/fpsyg.2026.1685494 (PMC12950689; doi:10.3389/fpsyg.2026.1685494)
Supplement: Supplementary file 1 [file Supplementary_file_1.docx]

Supplementary Material

Perceived Barriers and Learning Anxiety in Online Education Among Domestic and International Students

# Supplementary Data

In response to Reviewer 2’s comment that “Methods should keep essential details (sample, instruments, and analysis) while lengthy scale development steps should be moved to the Supplementary Materials,” we have provided the development procedures of the two instruments used in this study in the *Supplementary Material*.

# Scale Development

**Principles of scale preparation.** This study used two measures created for the study: the Online Learning Anxiety State Scale (hereafter referred to as the anxiety scale) and the Online Education Obstacles Scale (hereafter referred to as the obstacles scale). The anxiety scale was developed based on STAI, while the obstacle scale were developed based on relevant theories(TPACK) (Pelgrum &Plomp, 1993; Mishra & Koehler, 2006; Stuart et.al., 2009; Razaghi, 2014; Hoang et. al.,2019; Kouser & Popat, 2022) and previous measures (FLCAS), focusing on the five aspects of the obstacle to integrating technology and education mentioned above: teaching skills, internet/equipment, learning environment, interaction and technical communication, and insufficient material support(see table 1a for more details).

**Preparation and determination of anxiety scale.** Before the items of these two scales were decided, an open-ended questionnaire related to online learning was delivered, questions such as “Do you feel anxious about online courses compared to offline? Why?” and “When you encounter a problem you don't understand, is it easier to solve it online or offline? Why?” were included. These questions are aimed to figure out what barriers to the integration of technology may be associated with students' anxiety in online learning. The interview data serve as a reference for the development of items within both the online learning anxiety and obstacle scales. For instance, the observation that “network delays occur during online courses” was one of the findings gleaned from the previous investigation. This targeted description of the realities of online course obstacles profoundly considering the objectives of this study. Furthermore, this focused scope also enhances the “state” quality of anxiety, which are significant content restrictions.

After successfully collecting 35 valid questionnaires—comprising 17 domestic Chinese students and 18 international students—in-depth interviews were conducted with 5 students (3 domestic Chinese students and 2 international students). From these, initial versions of two measures were created: a 30-item anxiety scale and a 38-item obstacle scale.

In the second step, two domestic undergraduate students, five international students of different languages and two questionnaire assessment experts were invited to review and revise the first draft of the scale. The main task at this stage was to judge (a) content and face validity of scale items, (b) whether additional items were needed, and (c) whether question wording was clear, accurate, and easy to understand. Combined with the opinion of the assessment experts, we meticulously evaluated each question for its direct relevance to the survey’s objectives, utilizing the principles of content validity and face validity as benchmarks. Consequently, we eliminated items that were deemed either irrelevant or unhelpful in the context of information gathering.

Through this step, a total of 13 items on the anxiety scale were identified, including eight items scored positively. (e.g., Online courses, due to the lack of study partners, I feel more challenging to solve problems.) and five reverse-scored items (e.g., Since everyone is taking classes online, there is no need to panic.). Concurrently, to enhance the accessibility of the response scale for international students, all items on the anxiety scale have been translated into five foreign languages in addition to Chinese. Following the development of the scale items in Chinese, the full set of items was translated into Arabic, English, French, Korean and Thai (All of the versions are translated through Google and Youdao Translation first, and for each of these five languages, a native speaker proficient in Chinese was invited to double check the translations for accuracy. e.g. the Arabic, Thai and French version was respectively checked by high level Chinese L2 who comes from The Republic of Tunisia, Thailand and Cameroon. English and Korean version were checked by the author of this article).

The rationale behind this languages design is to take into account the common or official languages of most international students in China, and the scope of international students within the capacity of researchers. Items on the obstacle scale are presented in these same languages mentioned above, and its process is the same as the anxiety scale.

The difference between our Online Anxiety Scale and the STAI is that we have added a topic description about online courses to narrow the focus of the survey. This means that the term 'online classes' is used in place of the original STAI’s more general wording. For example, in the original STAI, one item is described as “ I feel worried.” While in our anxiety scale, the item has been changed to: “In online classes, delays in the internet make me feel worried that I might miss important knowledge points.” This revision is based on the results of previous surveys.

Establishment and determination of obstacle scale. The process of preliminary investigation and item validity of the obstacle scale is the same as that of the anxiety scale. After the second step we determined 28 items of obstacle scale. However, unlike the preparation of the anxiety scale, the item determination for the obstacle scale is more complex, as it requires an exploration of the dimensions that hinder the integration of online courses and technology. The primary distinction lies in our employment of statistical analysis to assess the structural validity of the obstacle scale. This paper primarily analyzes the structural validity of the obstacle scale from two perspectives: item analysis and exploratory factor analysis. Based on the analysis results, the dimensions of the scale are delineated, and the items within the corresponding dimensions are streamlined and refined. The revision and analysis of the integration disorder scale are elaborated upon in the subsequent section. The formal item determination process for the obstacle scale will be detailed below in “Analysis” part.

## Procedure

Data collection for the study occurred from April - June 2022. This time period was chosen to collect data because the Chinese government’s policy on nucleic acid testing changed significantly in April 2022, which meant that international students would have the opportunity to return to Chinese universities and in-person instruction would fully resume.

## Measures

The item composition of the questionnaire. In the formal study, a total of 60 questionnaire items were issued, encompassing both the online anxiety scale and obstacle scale. Among these, there were 6 questions pertaining to basic information, 28 items for obstacle questions, 13 items for anxiety questions and other 11 questions about offline courses. The offline course questions are designed to increase the reliability and validity of the questionnaire, ensure that respondents take the questionnaire seriously, and reduce the number of invalid questionnaires.

Experiences and Views of Online Learning. The survey included a set of open-ended questions primarily designed to elicit basic information about students’ experiences with and views of online learning, including students’ onset year and length of online learning, overall evaluation of and feelings about online learning (e.g., what kinds of courses are most suitable for online learning), and when students felt that they started to adapt to online courses.

Online Learning Barriers Ranking. A ranking question “I think there are some problems with online courses, and I will sort them in order of magnitude. (The first one is the most problematic)” was designed at the end of this questionnaire. For this question, students were asked to rank their perceptions of the relative gravity of various issues inherent to online courses.

Obstacles to Online Learning. The Online Education Obstacles Scale contained 28 items and incorporated five dimensions: skill factors, technological factors (e.g., issues with internet access), teaching materials, environmental factors (e.g., issues with learning atmosphere), and interactional factors (e.g., questions to or from the instructor); 15 of the items were reverse-scored (e.g., “It is very convenient to submit assignments for online courses.”).

# Measures

**The item composition of the questionnaire.** In the formal study, a total of 60 questionnaire items were issued, encompassing both the online anxiety scale and obstacle scale. Among these, there were 6 questions pertaining to basic information, 28 items for obstacle questions, 13 items for anxiety questions and other 11 questions about offline courses. The offline course questions are designed to increase the reliability and validity of the questionnaire, ensure that respondents take the questionnaire seriously, and reduce the number of invalid questionnaires.

**Experiences and Views of Online Learning.** The survey included a set of open-ended questions primarily designed to elicit basic information about students’ experiences with and views of online learning, including students’ onset year and length of online learning, overall evaluation of and feelings about online learning (e.g., what kinds of courses are most suitable for online learning), and when students felt that they started to adapt to online courses.

**Online Learning Barriers Ranking.** A ranking question “I think there are some problems with online courses, and I will sort them in order of magnitude. (The first one is the most problematic)” was designed at the end of this questionnaire. For this question, students were asked to rank their perceptions of the relative gravity of various issues inherent to online courses.

Obstacles to Online Learning. The Online Education Obstacles Scale contained 28 items and incorporated five dimensions: skill factors, technological factors (e.g., issues with internet access), teaching materials, environmental factors (e.g., issues with learning atmosphere), and interactional factors (e.g., questions to or from the instructor); 15 of the items were reverse-scored (e.g., “It is very convenient to submit assignments for online courses.”)

# Tables

**Table 1a Factor load matrix after rotation(obstacle scale)**

| Item | Factor 1(skill) | Factor 2(internet/equipment) | Factor 3(environmental) | Factor 4(material) | | Factor 5(interactional) |
| --- | --- | --- | --- | --- | --- | --- |
| Q16 | 0.903 |  |  |  |  | |
| Q7 | 0.828 |  |  |  |  | |
| Q10 | 0.823 |  |  |  |  | |
| Q11 | 0.636 |  |  |  |  | |
| Q28 | 0.618 |  |  |  |  | |
| Q48 |  | 0.863 |  |  |  | |
| Q42 |  | 0.816 |  |  |  | |
| Q47 |  | 0.784 |  |  |  | |
| Q41 |  | 0.761 |  |  |  | |
| Q20 |  | 0.507 |  |  |  | |
| Q44 |  |  | 0.931 |  |  | |
| Q45 |  |  | 0.645 |  |  | |
| Q35 |  |  | 0.536 |  |  | |
| Q12 |  |  | 0.517 |  |  | |
| Q52 |  |  |  | 0.788 |  | |
| Q51 |  |  |  | 0.763 |  | |
| Q19 |  |  |  | 0.574 |  | |
| Q46 |  |  |  |  | 0.733 | |
| Q23 |  |  |  |  | 0.691 | |
| Q37 |  |  |  |  | 0.566 | |

**Table 1b Pearson correlation test results between the Obstacle Scale and the Anxiety Scale(EFA)**

|  | Variables | 1 | 2 | 3 | 4 | 5 | 6 |
| --- | --- | --- | --- | --- | --- | --- | --- |
| 1 | skill factors | 1 |  |  |  |  |  |
| 2 | internet/equipment factors | .197** | 1 |  |  |  |  |
| 3 | environmental factors | .509** | .136* | 1 |  |  |  |
| 4 | teaching materials | 0.099 | .547** | 0.069 | 1 |  |  |
| 5 | interactional factors | .238** | -.254** | .167* | -.280** | 1 |  |
| 6 | Anxiety | .483** | .545** | .498** | .360** | 0.050 | 1 |

**. indicates that the correlation is significant at the 0.01 level(2-tailed)

*. indicates significant correlation at the 0.05 level(2-tailed)

**Table 1c Coefficientsa estimates of the model with five predictors and online anxiety as an outcome variable(EFA)**

| Model | | Unstandardized Coefficients | |  | Standardized Coefficients | t | Sig. |
| --- | --- | --- | --- | --- | --- | --- | --- |
|  |  | B | Std. error |  | Beta |  |  |
|  | 1 (Constant) | 17.080 | 2.333 |  |  | 7.321 | .000 |
|  | Skill factors | .277 | .076 |  | .210 | 3.634 | .000 |
|  | Internet/Equipment | .519 | .072 |  | .422 | 7.173 | .000 |
|  | environmental | .530 | .095 |  | .312 | 5.605 | .000 |
|  | Teaching materials | .285 | .148 |  | .111 | 1.917 | .057 |
|  | Interactional | .291 | .178 |  | .087 | 1.641 | .102 |

1. Dependent Variable: Anxiety

**Table 2 a Ranking the importance of obstacles among domestic Chinese and international students**

| Nationality | Mean(Chinese students=132) | | | Mean(International students=80) | t | Sig. (2-tailed) |
| --- | --- | --- | --- | --- | --- | --- |
| skill factors | | | 17.38 | 18.14 | -1.226 | 0.222 |
| Internet/Equipment | | | 10.73 | 15.06 | -6.468 | 0.000 |
| environmental factors | | | 14.26 | 14.95 | -1.440 | 0.151 |
| interactional | | | 10.73 | 10.11 | 2.558 | 0.011 |
| teaching materials | | | 6.12 | 6.74 | -1.779 | 0.078 |
| IP | | Mean(Domestic students=155) | | Mean(Oversea students=57) | t | Sig. (2-tailed) |
| skill factors | | | 17.19 | 18.95 | -2.624 | 0.009 |
| Internet/Equipment | | | 10.97 | 16.16 | -6.649 | 0.000 |
| environmental factors | | | 14.04 | 15.82 | -3.478 | 0.001 |
| teaching materials | | | 6.13 | 6.96 | -2.036 | 0.045 |
| interactional | | | 10.58 | 10.26 | 1.194 | 0.234 |

**Table 3a Correlation analyses for relations between obstacles and anxiety separated by domestic / international status** (N*_Chinese_*=132, N*_inernational_*=80)

|  | Chinese | 1 | 2 | 3 | 4 | 5 | 6 | 7 |
| --- | --- | --- | --- | --- | --- | --- | --- | --- |
| 1 | Anxiety | 1 |  |  |  |  |  |  |
| 2 | Teacher skill | .597^**^ | 1 |  |  |  |  |  |
| 3 | Internet/Equipment | .333^**^ | 0.011 | 1 |  |  |  |  |
| 4 | environmental | .577^**^ | .520^**^ | -0.006 | 1 |  |  |  |
| 5 | Materials | .261^**^ | -0.024 | .693^**^ | -0.001 | 1 |  |  |
| 6 | Interaction | 0.152 | .297^**^ | -.410^**^ | .334^**^ | -.350^**^ | 1 |  |
|  | International | 1 | 2 | 3 | 4 | 5 | 6 | 7 |
| 1 | Anxiety | 1 |  |  |  |  |  |  |
| 2 | Teacher skill | .322^**^ | 1 |  |  |  |  |  |
| 3 | Intent/Equipment | .649^**^ | .349^**^ | 1 |  |  |  |  |
| 4 | Environmental | .371^**^ | .482^**^ | .225^*^ | 1 |  |  |  |
| 5 | Materials | .422^**^ | 0.215 | .452^**^ | 0.129 | 1 |  |  |
| 6 | Interaction | 0.036 | 0.201 | -0.019 | -0.037 | -0.178 | 1 |  |

**. indicates that the correlation is significant at the 0.01 level(2-tailed)

*. indicates significant correlation at the 0.05 level(2-tailed)
